# Overexpression of the WRKY transcription factor gene *NtWRKY65* enhances salt tolerance in tobacco (*Nicotiana tabacum*)

**DOI:** 10.1186/s12870-024-04966-0

**Published:** 2024-04-24

**Authors:** Xiaoquan Zhang, Yaxuan Zhang, Man Li, Hongfang Jia, Fengjie Wei, Zongliang Xia, Xuelin Zhang, Jianbo Chang, Zhaojun Wang

**Affiliations:** 1 College of Tobacco Science, Henan Agricultural University, Zhengzhou, 450046 China; 2 Sanmenxia Branch of Henan Provincial Tobacco Corporation, Sanmenxia, 472000 China; 3 College of Agronomy, Henan Agricultural University, State Key Laboratory of Wheat and Maize Crop Science, Zhengzhou, 450046 China

**Keywords:** Salt tolerance, NtWRKY65, Tobacco plants, WRKY family transcription factor

## Abstract

**Background:**

Salt stress severely inhibits plant growth, and the WRKY family transcription factors play important roles in salt stress resistance. In this study, we aimed to characterize the role of tobacco (*Nicotiana tabacum*) *NtWRKY65* transcription factor gene in salinity tolerance.

**Results:**

This study characterized the role of tobacco (*Nicotiana tabacum*) *NtWRKY65* transcription factor gene in salinity tolerance using four *NtWRKY65* overexpression lines. NtWRKY65 is localized to the nucleus, has transactivation activity, and is upregulated by NaCl treatment. Salinity treatment resulted in the overexpressing transgenic tobacco lines generating significantly longer roots, with larger leaf area, higher fresh weight, and greater chlorophyll content than those of wild type (WT) plants. Moreover, the overexpressing lines showed elevated antioxidant enzyme activity, reduced malondialdehyde content, and leaf electrolyte leakage. In addition, the Na^+^ content significantly decreased, and the K^+^/Na^+^ ratio was increased in the *NtWRKY65* overexpression lines compared to those in the WT. These results suggest that *NtWRKY65* overexpression enhances salinity tolerance in transgenic plants. RNA-Seq analysis of the *NtWRKY65* overexpressing and WT plants revealed that *NtWRKY65* might regulate the expression of genes involved in the salt stress response, including cell wall component metabolism, osmotic stress response, cellular oxidant detoxification, protein phosphorylation, and the auxin signaling pathway. These results were consistent with the morphological and physiological data. These findings indicate that *NtWRKY65* overexpression confers enhanced salinity tolerance.

**Conclusions:**

Our results indicated that NtWRKY65 is a critical regulator of salinity tolerance in tobacco plants.

**Supplementary Information:**

The online version contains supplementary material available at 10.1186/s12870-024-04966-0.

## Background

Salt stress is one of the most fatal abiotic stresses influencing plant development and leads to growth inhibition, senescence, and death [1]. Salt stress is primarily caused by soil salinization, and its severity is exacerbated owing to irrational irrigation, frequent floods due to climate change, and inadequate drainage systems [2]. Therefore, elucidating the mechanisms underlying plant salt tolerance and applying this knowledge to breed salt-resistant cultivars are crucial.

Salt stress induces plant injury through two mechanisms: first, high salt concentrations can cause water deficit and osmotic stress; second, salt ions are toxic to plants [3]. Water deficit coping mechanisms include stomatal closure, which hampers gas exchange [4], and impairs photosynthetic efficiency [5, 6]. Plants absorb and accumulate salt ions (mainly Na^+^) at relatively high concentrations under salt stress. Salt ions are toxic and can inhibit the activity of many enzymes [7]. Over-accumulation of Na^+^ can inhibit K^+^ uptake, resulting in ionic imbalance [8, 9]. In addition to these physiological and biochemical changes, the reactive oxygen species (ROS) content in plant cells dramatically increases. This may cause lipid peroxidation, membrane deterioration, and DNA and protein damage [10, 11].

Plants have evolved several mechanisms to cope with salt stress [2]. Sodium ions can accumulate at a relatively high concentration in the cytosol; therefore, plant cells must transport them out of the cell [12] or store them in the vacuolar compartment [13]. This reduces the salt ion concentration to a harmless level in the cytoplasm and helps alleviate the osmotic stress [14]. Another effective method of osmotic adjustment is the biosynthesis and accumulation of osmolytes in the cytosol [15]. Osmolytes are low molecular weight organic solutes, such as carbohydrates [16] and nitrogen compounds [17, 18], that are not harmful to enzymes and other cellular structures at high concentrations. In the face of toxic accumulation of ROS, the antioxidant system is mobilized. This system comprises two major categories: ROS-scavenging enzymes, such as superoxide dismutase (SOD) [19], catalase (CAT) [20], and peroxidase (POD) [21], and non-enzymatic antioxidants, such as ascorbate, glutathione, and phenolic compounds [22]. Phytohormones also influence plant salt resistance. Abscisic acid (ABA) may mediate salinity signals to regulate stomatal closure. It coordinates with Ca^2+^ signals to regulate Na^+^ compartmentalization and K^+^ import, and integrates and regulates the signaling cascades of other phytohormones involved in salt stress resistance, such as brassinolide, auxin, gibberellin, and cytokinin [23]. Salt response-related genes encode signal-conducting proteins [24, 25], ion transporters [12, 13, 26], metabolic enzymes [27, 28], and many transcription factor genes, such as the bHLH, bZIP, MYB, NAC, and ZFP family genes [29] that play important roles in regulating the expression of many salt stress-responsive genes.

WRKY transcription factors belong to a large family that contains the highly conserved heptapeptide WRKYGQK in the N-terminus of the DNA-binding domain. They specifically bind to the W-box cis-element (T)TGAC(C/T) in the promoters of target genes. Many WRKY transcription factors are involved in the salt stress response. For example, *Arabidopsis* AtWRKY46 promotes lateral root development under salt stress [30]. In rice, OsWRKY54 regulates the expression of the *OsHKT1;5* gene by directly binding to the W-box motif in the promoter and enhancing salt resistance [31]. Overexpression of the transcriptional repressor OsWRKY50 also increases salt stress tolerance [32]. In maize, ZmWRKY104 enhances salt stress resistance [33], whereas ZmWRKY86 negatively regulates salt stress tolerance [34].

In tobacco (*Nicotiana tabacum*), 164 *WRKY* transcription factor genes were identified [35]. Our preliminary results showed that *NtWRKY65* was upregulated by ABA treatment. This suggests that it may be involved in abiotic stress resistance. This study further characterized the function of *NtWRKY65* in response to salt stress and compared the phenotypic and physiological differences between the wild type (WT) and overexpression lines under salt stress. We also performed a comparative transcriptome analysis between the WT and overexpression lines to further explore the function of *NtWRKY65*. Finally, the implications for understanding the function of *NtWRKY65* in the tobacco salt stress response are discussed.

## Results

### Cis-element and activity analyses of the *NtWRKY65* promoter

**Fig. 1:**
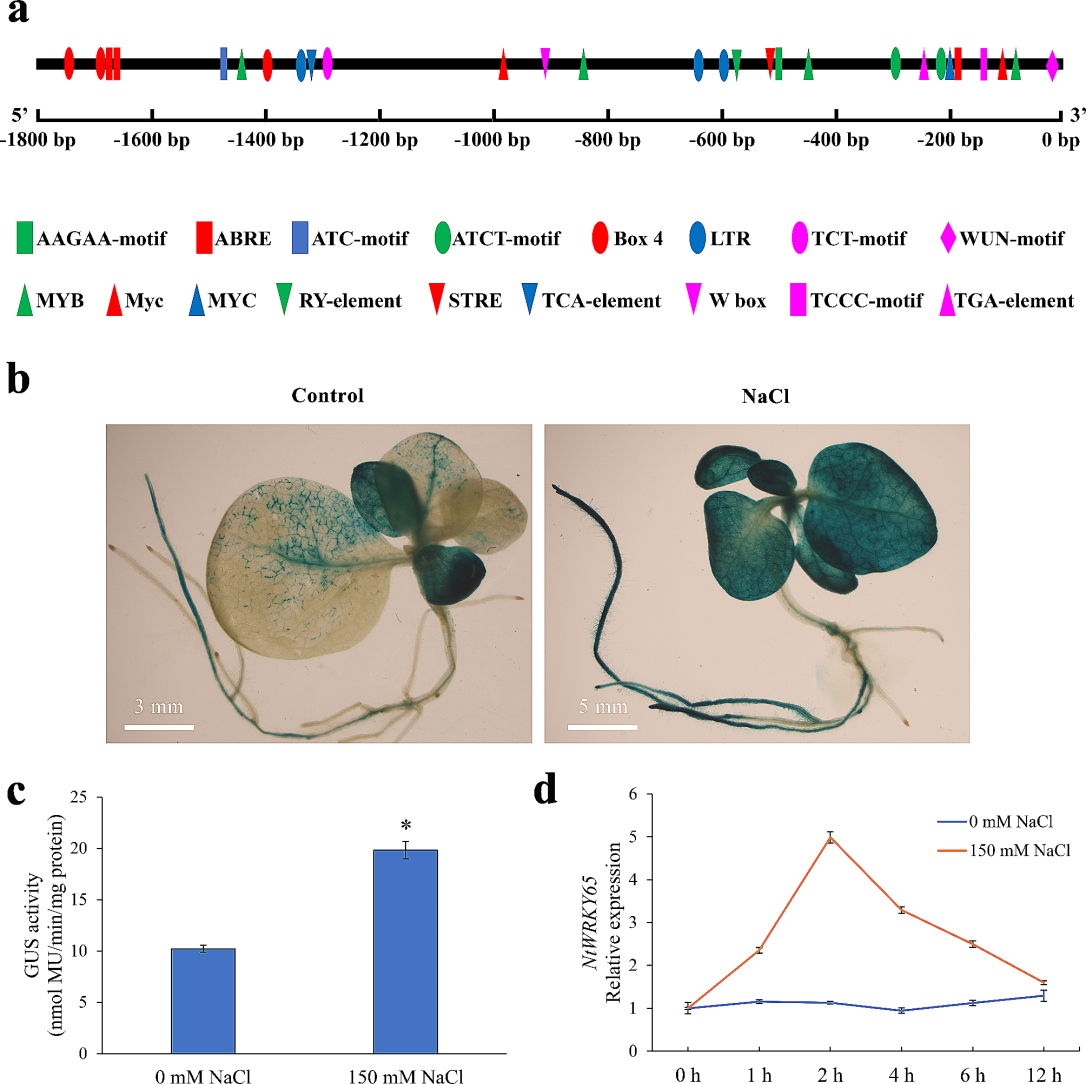
Cis-element and activity analysis of the *NtWRKY65* promoter. (**a**) Cis-elements of the *NtWRKY65* promoter. An 1800 bp fragment upstream of the start codon was analyzed. Core promoter elements such as the TATA-box and CAAT-box are not shown for clarity. (**b**) Activity analysis of the *NtWRKY65* promoter. Transgenic tobacco seedlings containing *NtWRKY65* promoter driven β-glucuronidase (GUS) recombinant stained after 0 mM and 150 mM NaCl treatment for two hours. (**c**) The GUS activity in transgenic tobacco containing *NtWRKY65* promoter driven GUS recombinant after 0 mM or 150 mM NaCl treatment for two hours. Columns and bars represent the mean and standard error of three seedlings, respectively. Asterisks indicate a significant difference at *P* < 0.05 by the Student’s *t*-test. (**d**) qRT-PCR analysis of *NtWRKY65* expression levels at different time intervals under 150 mM NaCl treatment. The relative expression was calculated by setting 0 h as 1

A 1.8 kbp fragment upstream of the start codon was downloaded from the reference genomic sequence of tobacco cultivar K326 [36] and queried using the PlantCARE database (http://bioinformatics.psb.ugent.be/webtools/plantcare/html/). As shown in Fig. 1a and Additional File 1: Table S1, the identified cis-elements were mainly involved in responses to light (e.g., ACT-motif, ATCT-motif, Box 4, TCCC-motif, TCT-motif), hormones, and abiotic stress (e.g., ABRE, TCA-element, TGA-element, LTR, MYB, W-box). This indicated that *NtWRKY65* expression is regulated by these factors.

To examine the spatial expression pattern of *NtWRKY65* and its promoter activity, we constructed an *NtWRKY65* promoter-driven GUS vector and used it to transform tobacco plants. Histochemical assays of *NtWRKY65* promoter-driven GUS transgenic seedlings under normal growth conditions showed that the young leaves and axial roots were primarily stained (Fig. 1b). After treating the transgenic seedlings with 150 mM NaCl, the most deeply stained parts were the young leaves and roots, followed by mature leaves (Fig. 1b). Furthermore, the enzymatic activity of GUS was significantly higher after the 150 mM NaCl treatment than after the 0 mM NaCl treatment (Fig. 1c). These results indicated that the NaCl treatment elevated *NtWRKY65* expression.

The response of *NtWRKY65* to NaCl treatment at different time intervals was examined using quantitative real-time PCR (qRT-PCR). Compared to the 0 mM NaCl treatment, the expression of *NtWRKY65* increased in response to the 150 mM NaCl treatment, reached a peak after 2 h of treatment, and decreased gradually (Fig. 1d).

### NtWRKY65 protein is nuclear-localized and has transactivation activity

**Fig. 2:**
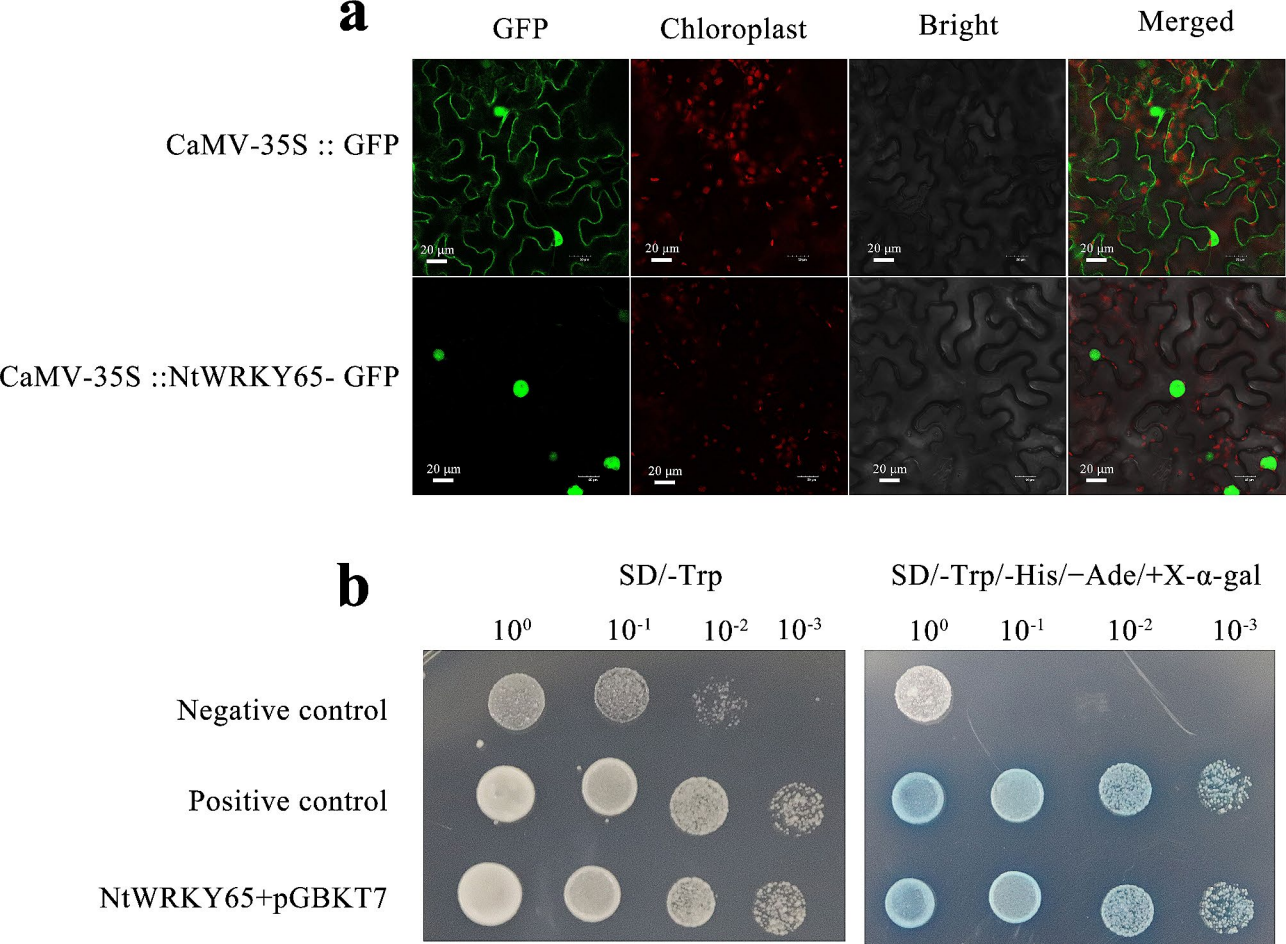
Subcellular localization and transactivation activity assay of NtWRKY65 protein. (**a**) Subcellular localization of NtWRKY65 protein in *Nicotiana benthamiana* epidermal cells. (**b**) Transactivation activity assay of the NtWRKY65 protein. The full-length coding sequence of *NtWRKY65* was inserted into the pGBKT7 vector, and transformed yeast clones were selected on SD/-Trp and SD/-Trp-His-Ade media. pGBKT7-53 was used as the positive control. Empty pGBKT7 was used as the negative control

Subcellular localization of the NtWRKY65 protein was determined by fusing the CDS of the *NtWRKY65* gene to a green fluorescent protein gene driven by the CaMV-35 S promoter, and transiently expressed it in *Nicotiana benthamiana* leaves. The fluorescent signal in the CaMV-35 S::GFP control vector transformed leaves was distributed throughout the cell (Fig. 2a), whereas the green fluorescent signal in the CaMV-35 S::GFP-NtWRKY65 vector transformed leaves was observed only in the nucleus. This suggests that NtWRKY65 is a nuclear localized protein.

We further examined whether NtWRKY65 possesses transcriptional activity. The CDS of the *NtWRKY65* gene was fused with the coding sequence of the DNA-binding domain of the *Saccharomyces cerevisiae* transcription activating protein gene *GAL4* to obtain the pGBKT7-NtWRKY65 vector; the pGBKT7-53 (encodes a fusion of murine p53 protein and the GAL4 DNA-binding domain) and pGBKT7 empty vectors were used as the positive and negative controls, respectively. The three plasmids were separately transformed into the AH109 yeast strain and inoculated onto SD/-Trp, and SD/-Trp/-His/-Ade/X-α-gal medium. Transformants containing pGBKT7-NtWRKY65 grew well on SD/-Trp and SD/-Trp/-His/-Ade medium, and displayed a blue color in the presence of X-α-gal. The results were comparable to those of positive control. By contrast, transformants containing pGBKT7 grew on the SD/-Trp medium, but not on the SD/-Trp /-His/-Ade medium, and did not display a blue color in the presence of X-α-gal (Fig. 2b). This indicated that NtWRKY65 had transcriptional activation activity.

### *NtWRKY65* overexpression enhanced the salt stress tolerance of *N. tabacum*

We constructed four *NtWRKY65-*overexpressing transgenic lines using the tobacco cultivar K326, namely OE2, OE5, OE8, and OE11. qRT-PCR analysis showed that *NtWRKY65* expression significantly increased in the four lines compared to that in WT (Additional File 2: Fig. S1).

**Fig. 3:**
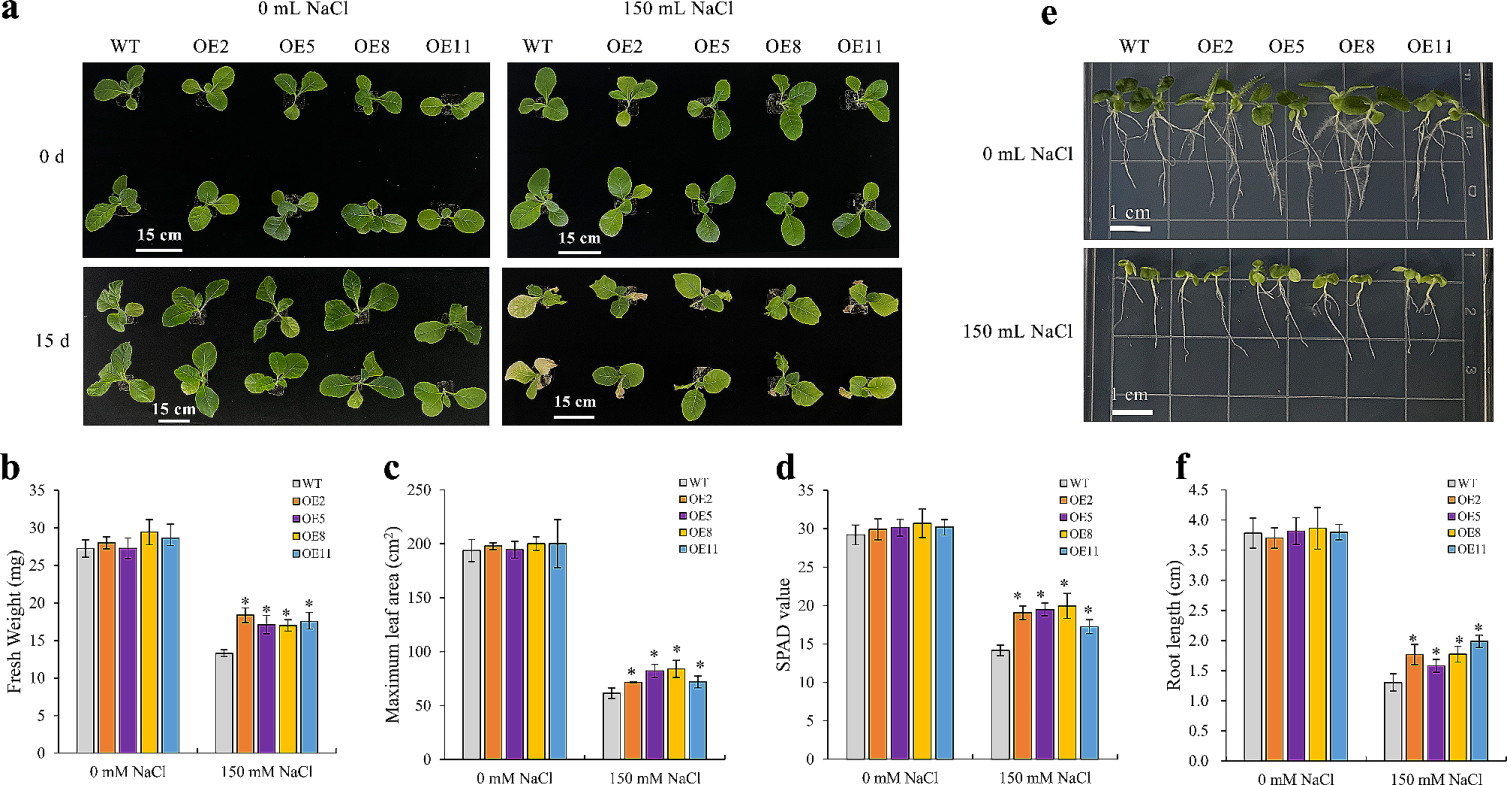
Salt stress tolerance of wild type (WT) and *NtWRKY65* overexpression seedlings. (**a**) Growth status of wild type (WT) and *NtWRKY65* overexpression seedlings treated with 0 mM NaCl and 150 mM NaCl for 14 d. Scale bars are 15 cm. (**b**) Fresh weight, (**c**) maximum leaf area, (**d**) SPAD value, (**e**) root phenotype, and (**f**) root length of WT and *NtWRKY65* overexpression seedlings treated with 0 mM and 150 mM NaCl for 14 d. Columns and bars represent the means and standard errors of three seedlings, respectively. Significant differences between the WT and overexpression lines were determined using Student’s *t*-test, * *P* < 0.05, ** *P* < 0.01

Seedlings of the WT and four overexpression lines grown in nutrient bowls were watered with Hoagland’s solution [37] containing 0 or 150 mM NaCl for 14 d. There was no obvious difference between the growth of WT and overexpression seedlings under the 0 mM NaCl treatment. However, the growth of the WT and overexpression lines was delayed for seedlings treated with 150 mM NaCl, but the growth status of the four overexpression lines was much better than that of WT. As shown in Fig. 3a, under the 150 mM NaCl treatment, almost all the leaves of WT plants turned yellow, whereas only the lower leaves of overexpression plants turned slightly yellow, the fresh weight (Fig. 3b) and maximum leaf areas (Fig. 3c) of all the four overexpression lines were significantly higher than those of WT plants. SPAD values indicated that the accumulation of chlorophyll was significantly higher in all the four overexpression line than in WT plants (Fig. 3d). Furthermore, the root length of the overexpression lines was greater than that of the WT plants under the 150 mM NaCl treatment (Fig. 3e, f).

### *NtWRKY65* overexpression improves tobacco antioxidant ability

**Fig. 4:**
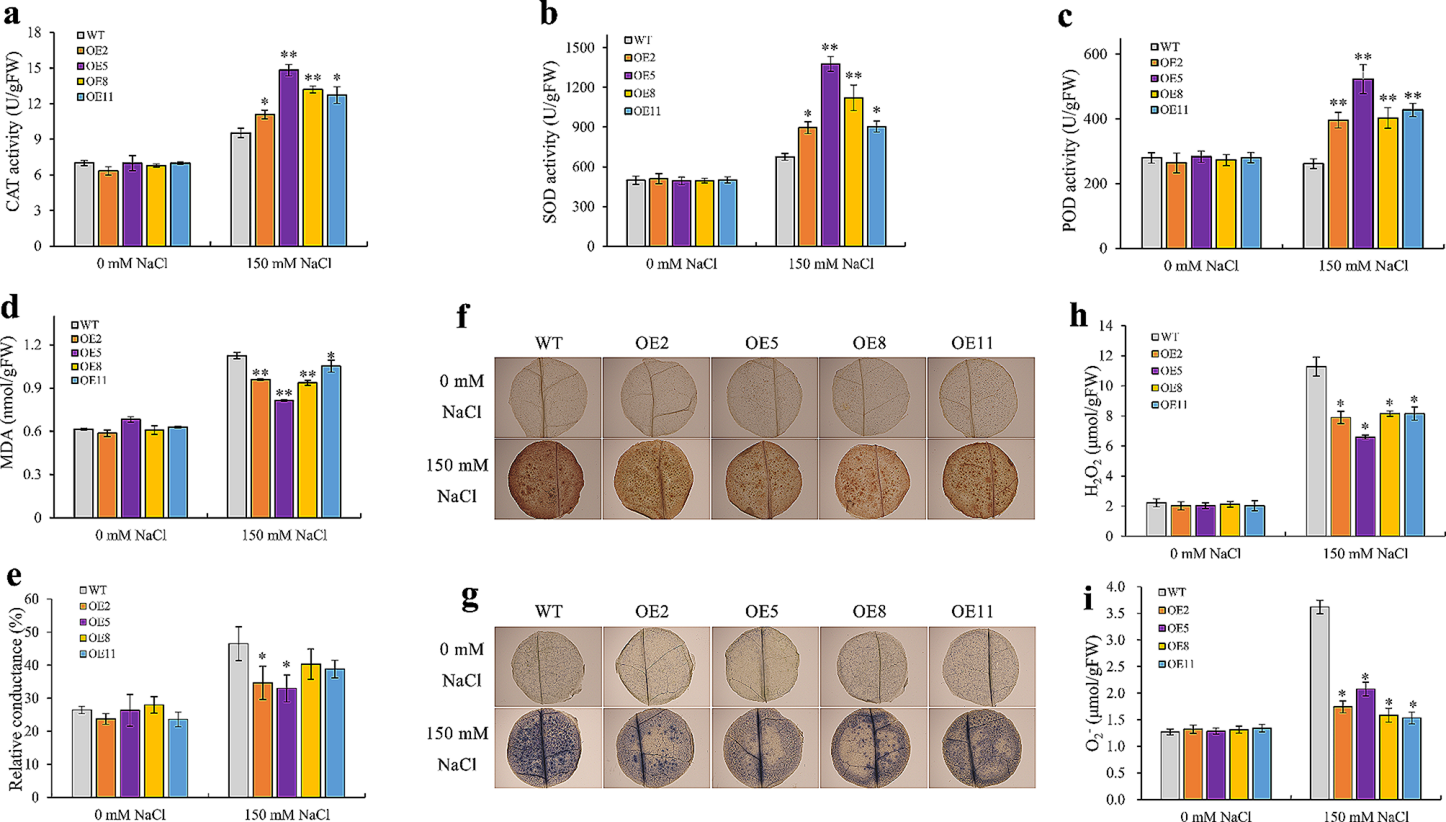
Antioxidant assay of wild tpe (WT) and *NtWRKY65* overexpression lines. Activity of (**a**) CAT, (**b**) SOD, (**c**) POD, and the levels of (**d**) MDA and (**e**) leaf relative conductance analyzed after treatment using 0 mM NaCl and 150 mM NaCl. Leaf ROS accumulation was visualized by (**f**) DAB and (g) NBT staining. Content of (h) H_2_O_2_ and (i) O_2_^−^ after 0 mM NaCl and 150 mM NaCl treatment. Columns and bars represent the means and standard errors of three seedlings, respectively. Significant differences between the WT and overexpression lines were determined using Student’s *t*-test, * *P* < 0.05, ** *P* < 0.01

Salt stress significantly enhanced the ROS content, such as H_2_O_2_ and O_2_^−^, which damage cellular structures [11]. Antioxidant-related parameters were measured to further analyze the function of *NtWRKY65* in the salt stress response. There was no significant difference in the anti-oxidase activity, including that of CAT, POD, and SOD, between the WT and overexpression lines under the 0 mM NaCl treatment. Meanwhile, the activities of CAT, SOD, and POD were significantly enhanced in the overexpression lines under the 150 mM NaCl treatment compared to those in the WT (Fig. 4a-c).

Malondialdehyde (MDA) content and leaf relative conductance, which are indicators of membrane lipid peroxidation [11], were comparable in the WT and overexpression lines under the 0 mM NaCl treatment. However, under the 150 mM NaCl treatment, these parameters were significantly lower in overexpression lines than in the WT plants (Fig. 4d, e).

To further confirm the ROS scavenging ability of the *NtWRKY65* overexpression lines, diaminobenzidine tetrahydrochloride (DAB) and nitroblue tetrazolium (NBT) staining methods were used to determine the levels of H_2_O_2_ and O_2_^−^, respectively. There was no significant difference in DAB and NBT staining results between the WT plants and *NtWRKY65* overexpression plants under the 0 mM NaCl treatment. However, the DAB (Fig. 4f) and NBT (Fig. 4g) staining intensity of WT leaves was much stronger than that of the leaves from the *NtWRKY65* overexpressing plants under the 150 mM NaCl treatment. The H_2_O_2_ and O_2_^−^ contents in the leaves of the overexpressing and WT plants under the 0 mM NaCl treatment did not differ significantly, but were significantly lower in the *NtWRKY65* overexpressing plants than in the WT under the 150 mM NaCl treatment (Fig. 4h, i). This observation indicated that ROS accumulation was lower in the four *NtWRKY65* overexpression lines than in the WT plants.

### *NtWRKY65* overexpression increases the tobacco K^+^/Na^+^ ratio

**Fig. 5:**
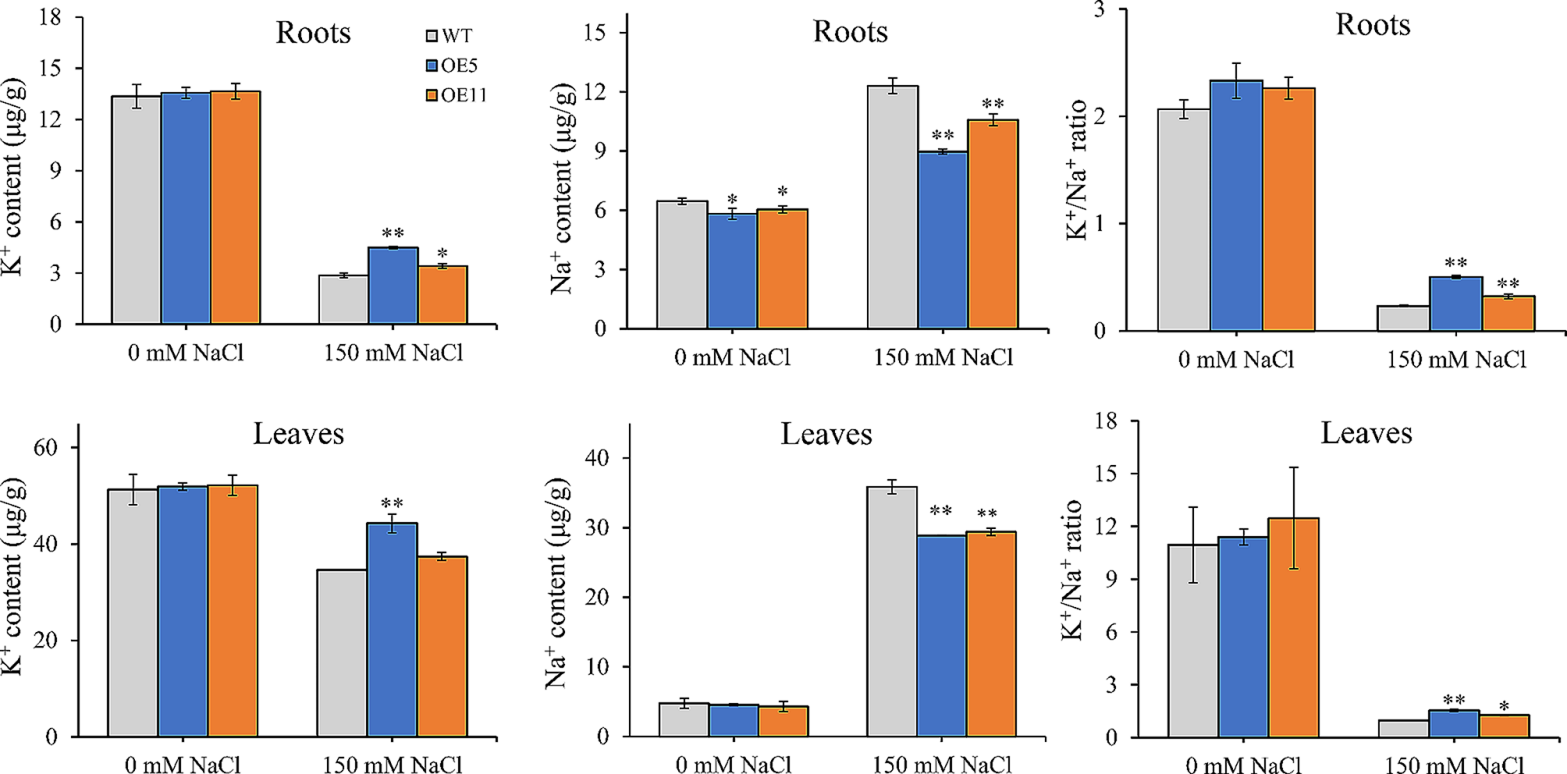
Na^+^ and K^+^ contents in wild type (WT) and *NtWRKY65* overexpression lines under NaCl treatment. Columns and bars represent the means and standard errors of three seedlings, respectively. Significant differences between the WT and overexpression lines were determined using Student’s *t*-test, * *P* < 0.05, ** *P* < 0.01

There was no significant difference in the Na^+^ and K^+^ contents between WT and *NtWRKY65* overexpression lines under the 0 mM NaCl treatment, both in the root and leaves. However, the K^+^ content in overexpression lines was significantly higher under the 150 mM NaCl treatment compared with that in WT plants, while the Na^+^ content was significantly lower in overexpression lines than that in WT plants. This resulted in a higher K^+^/Na^+^ ratio in overexpression lines compared to that in WT plants, with roots and leaves showing similar patterns (Fig. 5).

### RNA-seq analysis of *NtWRKY65* overexpressed transgenic tobacco

A comparative transcriptome analysis was performed between WT and OE11 under the 150 mM NaCl treatment, and the results were compared with those of the 0 mM NaCl control. Approximately 40 million clean reads were obtained for each sample, with 93.75–96.61% of clean reads successfully mapped to the reference genome [36], and over 89.76% uniquely mapped (Additional File 3: Table S2). Pearson’s correlation analysis based on gene expression showed that biological replicates were more highly correlated within samples than between samples (Additional File 2: Fig. S2). These results indicated the high quality of the RNA-Seq data.

A comparison among different samples was performed by calculating the gene expression levels in each sample as fragments per kilobase of transcript per million fragments mapped (FPKM) [38], and differentially expressed genes (DEGs) were identified by setting a false discovery rate (FDR) < 0.01 and log_2_FoldChange > 1 or < − 1 as the threshold.

**Fig. 6:**
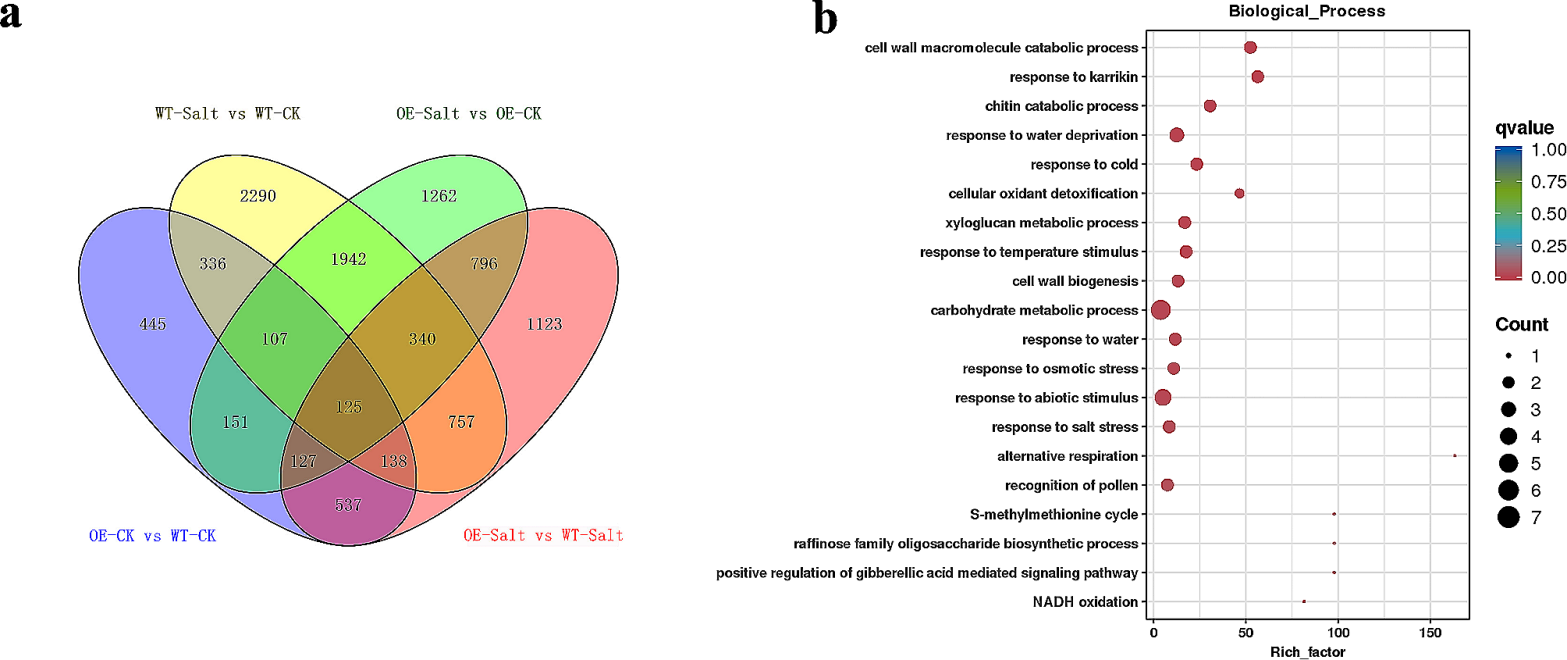
RNA-Seq analysis of the *NtWRKY65* overexpression line and wild type (WT) tobacco. (**a**) Venn diagram showing common and exclusive DEGs between OE11 and WT plants with or without NaCl treatment. (**b**) Top 20 most significantly enriched GO terms of the common DEGs between OE11-Salt vs. OE11-CK, WT-Salt vs. WT-CK, OE11-CK vs. WT-CK, and OE11- Salt vs. WT- Salt. -CK and -salt indicated the 0 mM NaCl and 150 mM NaCl treatment, respectively

DEGs were detected in four comparisons: (1) OE11 and WT plants under the 0 mM NaCl treatment, (2) OE11 and WT plants under the 150 mM NaCl treatment, (3) WT plants under the 0 and 150 mM NaCl treatment, and (4) OE11 plants under the 0 and 150 mM NaCl treatment. These analyses revealed 1966, 3943, 6035, and 4850 DEGs, respectively. Notably, 125 DEGs were common to all four comparisons (Fig. 6a). We hypothesize that these 125 DEGs represent candidate genes that are potentially regulated by *NtWRKY65* and are involved in the response to salt stress in tobacco.

The Gene Ontology (GO) enrichment analysis results of these 125 DEGs are listed in Additional File 4: Table S3, and the top 20 most significantly enriched GO terms are shown in Fig. 6b. Some putative salt stress response pathways, including cell wall component metabolism, response to osmotic stress, cellular oxidant detoxification, protein phosphorylation, and the auxin signaling pathway, were significantly enriched. The DEGs associated with these pathways are listed in Table 1.

**Table 1:** Putative salt stress responding pathways and genes regulated by NtWRKY65

Functional pathways	Gene_ID	FPKM				Gene annotation
		WT-CK	OE11-CK	WT-Salt	OE11-Salt	
Cell wall component metabolism	Nitab4.5_0001310g0010	18.23	42.04	1.7	21.02	Glycoside hydrolase, family 19
	Nitab4.5_0003207g0080	22.7	72.47	72.87	2124.31	Glycoside hydrolase, family 19
	Nitab4.5_0005898g0010	180.62	470.07	36.82	256.2	Glycoside hydrolase, family 19
	Nitab4.5_0001675g0070	0.67	3.66	3.66	11.26	Xyloglucan endotransglucosylase/hydrolase
	Nitab4.5_0002630g0010	2.8	26.52	23.25	77.17	Xyloglucan endotransglucosylase/hydrolase
Response to osmotic stress	Nitab4.5_0002712g0010	52.22	135.04	20.47	41.64	Wound stress protein, lipase/lipooxygenase
	Nitab4.5_0004173g0010	40.05	93.92	4.44	17.04	Wound stress protein, lipase/lipooxygenase
	Nitab4.5_0004173g0030	21.12	61.62	1.69	6.58	Wound stress protein, lipase/lipooxygenase
Cellular oxidant detoxification	Nitab4.5_0002823g0010	9.7	29.26	3.97	15.03	Glutathione S-transferase-like protein
	Nitab4.5_0002731g0010	0.9	5.4	4.49	17.05	Peroxidase
	Nitab4.5_0015635g0010	0.79	3.61	8.24	20.46	Peroxidase
Protein phosphorylation	Nitab4.5_0000038g0030	11.29	24.52	0.29	2.96	Cysteine-rich receptor-like protein kinase
	Nitab4.5_0010147g0020	37.86	93.32	2.76	37.68	Cysteine-rich receptor-like protein kinase
	Nitab4.5_0001223g0050	0.43	1.38	1.64	4.1	Serine_threonine-protein kinase receptor
	Nitab4.5_0004625g0010	1.5	3.08	0.3	1.11	Serine_threonine-protein kinase receptor
	Nitab4.5_0006529g0010	0.24	0.92	0.87	3.51	Serine_threonine kinase receptor
	Nitab4.5_0000057g0040	0.11	0.7	0.63	1.87	Receptor like kinase
Auxin signaling pathway	Nitab4.5_0000209g0290	8.37	19.72	2.27	7.47	Auxin responsive protein, AUX_IAA protein
	Nitab4.5_0004887g0040	6.53	24.96	1.42	5.28	Auxin responsive protein, AUX_IAA protein
	Nitab4.5_0001650g0130	14.75	35.33	6.19	18.44	Auxin responsive SAUR protein
Transcription factor	Nitab4.5_0000040g0110	2.78	10.92	11.60	25.93	NAC transcription factor
	Nitab4.5_0000048g0080	4.97	15.68	22.46	101.67	WRKY transcription factor
	Nitab4.5_0000381g0130	4.49	23.86	14.35	64.25	WRKY transcription factor
	Nitab4.5_0000569g0020	4.66	11.08	14.56	32.63	Zinc-finger protein, C2H2-type
	Nitab4.5_0000586g0140	0.80	2.69	3.56	12.88	Heat stress transcription factor
	Nitab4.5_0001472g0070	6.70	0.08	18.19	5.83	Basic-leucine zipper protein
	Nitab4.5_0002758g0040	4.93	20.64	32.69	155.25	WRKY transcription factor
	Nitab4.5_0007691g0010	0.75	3.77	2.39	11.99	WRKY transcription factor
	Nitab4.5_0012870g0010	10.60	31.87	4.80	17.38	WRKY transcription factor

*Note*: CK and -salt in the sample name indicate 0 mM NaCl and 150 mM NaCl treatments, respectively. FPKM, fragments per kilobase of transcript per million fragments mapped

Nine transcription factor genes were also identified among the 125 DEGs (Table 1), these genes may be involved in the salt stress resistance pathway mediated by *NtWRKY65*.

### qRT-PCR verification of putative salt stress responding genes regulated by *NtWRKY65*

**Fig. 7:**
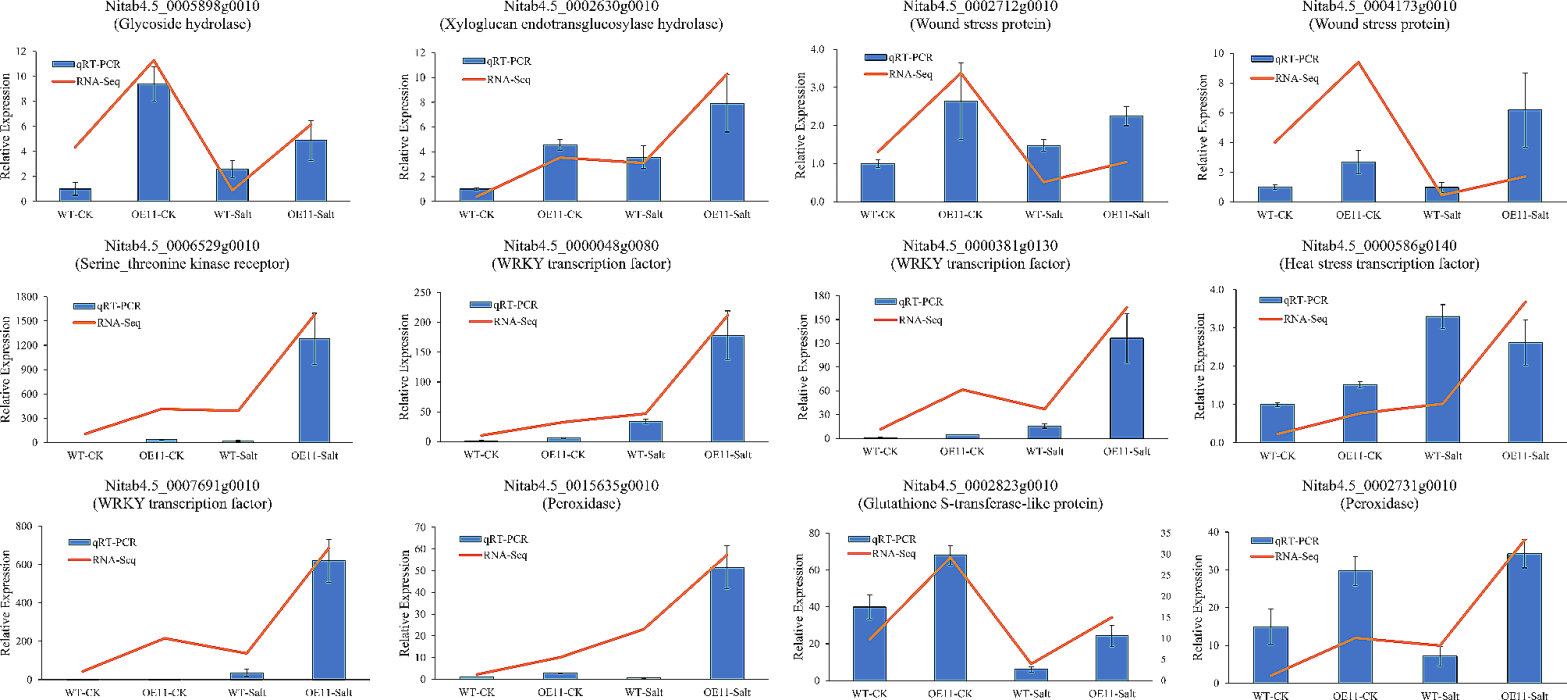
qRT-PCR confirmation of putative salt stress responding genes regulated by *NtWRKY65*. 12 DEGs were randomly selected from Table 1, brief annotation of each gene is shown in the brackets. Columns and bars represent the means and standard error of three seedlings, respectively. For each gene, the relative expression was calculated by setting 0 mM NaCl treated wild type plants as 1

To further corroborate the RNA-Seq results, 12 genes listed in Table 1 were randomly selected for qRT-PCR analysis, the primers used are listed in Additional file 5: Table S4. The results showed that although some quantitative differences exist, the expression pattern measured via qRT-PCR was similar to that obtained using the RNA-Seq results (Fig. 7), indicating the high quality of RNA-Seq results.

## Discussion

Salt stress can severely inhibit plant growth and development [1]. Thus identifying genes involved in plant salt stress resistance and elucidating their molecular functions is important for improving plant salt resistance breeding.

Some members of the WRKY family transcription factors are involved in plant salt stress responses [30–34], however, there are few reports on the relationship between WRKY transcription factors and salt stress resistance in *N. tabacum*, an important model plant for basic biological research. This study analyzed the function of the tobacco salt stress-responsive gene, *NtWRKY65*. Salt treatment upregulated the expression of *NtWRKY65* (Fig. 1). Salt stress resulted in a longer root length, greater leaf area, higher fresh weight, and increased chlorophyll content in *NtWRKY65* overexpression plants compared to WT plants (Fig. 3). This indicates that *NtWRKY65* plays a key role in tobacco salt stress resistance.

Cis-element analysis of the *NtWRKY65* gene promoter revealed that cis-elements were mainly involved in light response, hormonal regulation (ABA, auxin, and salicylic acid), and low-temperature response (Fig. 1a, Additional file 1: Table S1), among them, the number of light responsive cis-element was the largest. Consistent with our results, another study found that cis-elements associated with light response were the predominant type present in the promoter region of 21 salinity and drought responsive genes in *Arabidopsis* [39], this indicates that light may also influence salinity tolerance in tobacco through *NtWRKY65*. The presence of ABA, auxin, and salicylic acid responsive cis-elements in *NtWRKY65* promoter was expected because phytohormones play an important role in regulating plant salinity [23], thus, *NtWRKY65* may also be involved in the phytohormone mediated tobacco salinity tolerance. The presence of low-temperature response cis-elements suggests a cross-talk between the regulation network of salinity and low-temperature tolerance, which has been observed in previous studies [40], this cross-talk may be mediated by *NtWRKY65* in tobacco. Thus, the distribution pattern of the cis-elements in the promoter region provides important information for further research on the function of *NtWRKY65*.

We hypothesized that the function of *NtWRKY65* in salt stress resistance is related to root growth. A vital task for plants experiencing salt stress is coping with osmotic stress. Optimum root systems can support adequate water supply. Many studies have shown that root morphology reflects plant salt tolerance [41]. Our study found that the root growth of the WT and *NtWRKY65* overexpression lines was inhibited under salt stress; however, longer primary roots were found in the *NtWRKY65* overexpression lines (Fig. 3), in addition, the expression of *NtWRKY65* in the roots was significantly elevated under salt stress (Fig. 1). This indicates that *NtWRKY65* may modify root development under salt stress. Similarly, other WRKY transcription factors regulate root morphology in other plant species. For example, wheat *TaWRKY51* promotes lateral root formation [42], and *AtWRKY46* modulates the development of *Arabidopsis* lateral roots under osmotic or salt stress conditions [30]. Therefore, we speculate that these WRKY transcription factors probably share similar pathways for promoting root growth under salt stress, however, detailed studies are required to determine their molecular mechanisms.

We hypothesized that *NtWRKY65* promotes salt tolerance in tobacco through two physiological modes. First, *NtWRKY65* enhances the antioxidant capacity. The activity of antioxidizes (including CAT, POD, and SOD) was higher in *NtWRKY65* overexpression lines compared with WT plants under high salinity conditions (Fig. 4), whereas the MDA content and electrolyte leakage were significantly lower in *NtWRKY65* overexpression lines compared with WT plants. This suggests that the *NtWRKY65* overexpression lines were less damaged by ROS compared to the WT, consistent with the results obtained for other WRKY transcription factors involved in salt stress resistance [33, 43]. Second, *NtWRKY65* modulates the ion balance. Several studies have shown that in plants, salt stress increases the Na^+^ content and decreases the K^+^ content. Thus, the ability to maintain K^+^/Na^+^ homeostasis is an important index to evaluate salt stress tolerance [1]. This study found that the Na^+^ content in the roots and leaves of *NtWRKY65* overexpression lines was lower than that of WT plants under salt stress, while the K^+^ content in *NtWRKY65* overexpression lines was higher than that of WT plants. This led to a relatively higher K^+^/Na^+^ ratio, indicating that *NtWRKY65* helps maintain K^+^/Na^+^ homeostasis.

A comparative RNA-Seq analysis between *NtWRKY65* overexpression lines and WT plants with or without salinity treatment was performed. The results were consistent with the morphological and physiological data and revealed additional information regarding the function of *NtWRKY65* in response to salt stress.

In theory, DEGs common to both WT and OE11 under 0 mM versus 150 mM salt treatment are likely to be salinity responsive. Meanwhile, DEGs common to WT versus OE11 under both 0 mM and 150 mM treatments are likely to be regulated by *NtWRKY65*. Therefore, DEGs common across all four comparisons are likely to be functionally associated with *NtWRKY65* and play important roles in tobacco salt stress resistance. A total of 125 DEGs satisfied these criteria (Fig. 6a).

Among the 125 DEGs, we identified five genes related to cell wall component metabolism: three glycoside hydrolase genes and two xyloglucan endotransglucosylase/hydrolase genes (Table 1). Sequence analysis showed that the three glycoside hydrolase genes belonged to the glycoside hydrolase family 19 [44]. The function of this gene family is mainly associated with improved environmental stress resistance, and a member of this family in *Arabidopsis*, the gene *hot2*, has been found to prevent overaccumulation of Na^+^ [45]. Similarly, we found that Na^+^ content was reduced in *NtWRKY65* overexpression lines under the 150 mM NaCl treatment (Fig. 5), suggesting that the three genes we identified in this study also prevent the Na^+^ uptake in tobacco. Xyloglucan endotransglucosylase/hydrolase regulated cell wall extensibility and plasticity and maintained the appropriate strength. Many xyloglucan endotransglucosylase/hydrolase have been found to be involved in salinity tolerance [46], indicating that the two xyloglucan endotransglucosylase/hydrolase genes identified in this study may enhance the salinity tolerance of *NtWRKY65* overexpression plants.

Three wound stress responding proteins, annotated as lipase/lipoxygenase genes, were identified, which were functionally associated with osmotic stress response (Table 1). Previous studies showed that the *CaLOX1* gene in pepper [47] and *DkLOX3* gene in persimmon [48] could reduce H_2_O_2_ and O_2_^−^ accumulation and lipid peroxidation under salt stress, indicating that the enhanced ROS scavenging capacity of *NtWRKY65* overexpression lines (Fig. 4f-i) may partially be attributed to the lipase/lipoxygenase genes identified in this study. Another reason for the decreased ROS content in *NtWRKY65* overexpression lines was the elevated anti-oxidase activity (Fig. 4a-c). We identified a glutathione S-transferase gene and two peroxidase genes, which were up-regulated in OE11, the expression of these three genes was further confirmed using qRT-PCR (Fig. 7). These results indicate that *NtWRKY65* enhance the ROS scavenging ability of tobacco plants by modulating the expression of lipase/lipoxygenase, S-transferase, and peroxidase genes.

For the protein phosphorylation signal pathways, six protein kinase genes were identified (Table 1). Among them, two genes were annotated to coding cysteine-rich receptor-like protein kinase. Studies have reported the positive [49] or negative [50] role of cysteine-rich receptor-like protein kinase in plant salt stress resistance. Moreover, the W-box cis-elements, which are recognized by WRKY transcription factors, are enriched in the promoters of *Arabidopsis* cysteine-rich receptor-like protein kinase genes [51]. This suggests the possibility that the two cysteine-rich receptor-like protein kinase genes identified in this study may be under the regulation of *NtWRKY65* and may play a role in salt stress resistance. The other four protein kinase genes are G-type lectin serine/threonine-protein kinase receptor coding genes [52]; studies have reported the involvement of this gene subfamily in plant responses against salt stress. Ectopic expression of the *Glycine soja GsSRK* gene in *Arabidopsis* and *Medicago sativa* reduced the accumulation of Na^+^ and conferred transgenic plants with increased chlorophyll content, reduced ion leakage, increased plant height and increased root length under salt stress [53, 54], and overexpression of the *GmLecRlk* gene in *Glycine max* (L.) Merr. enhanced the ROS scavenging capacity in response to salt stress [55]. The results obtained in this study are consistent with those in the literature; thus, these G-type lectin serine/threonine-protein kinase receptor genes may have similar functions in tobacco during salt stress response.

Among the 125 key DEGs, three genes were auxin-responsive (Table 1). Auxin is a vital phytohormone that regulates root development [56]. This suggested that these three genes may regulate root development, resulting in a longer primary root in *NtWRKY65* overexpression lines (Fig. 3).

Nine transcription factor genes were identified (Table 1), five of which belonged to the WRKY family. Studies on *Arabidopsis* have found that three WRKY family genes (*AtWRKY18*, *AtWRKY40*, and *AtWRKY60*) work in coordination to enhance plant salt and osmotic stress resistance [57], and WRKY70 and WRKY54 cooperate as negative regulators of stomatal closure and osmotic stress tolerance [58]. Further verification is required to determine if the WRKY members identified in this study are also functionally associated with the salt stress resistance process.

## Conclusions

NtWRKY65 is located in the nucleus and exhibits transactivation activity. Its expression was up-regulated by NaCl treatment. The root length, leaf area, fresh weight, and chlorophyll content of *NtWRKY65* overexpression lines were higher than those of WT plants under salinity treatment. *NtWRKY65* overexpression lines showed elevated antioxidant enzyme activity, reduced malondialdehyde content, and leaf electrolyte leakage compared to the WT plants following NaCl treatment. In addition, the Na^+^ content significantly decreased, and the K^+^/Na^+^ ratio improved in the overexpression lines. RNA-Seq analysis showed that *NtWRKY65* may regulate the expression of salt stress-responsive genes. The results from this study may aid further research on *NtWRKY65* and plant salinity tolerance.

## Methods

### Plant materials and growth conditions

The common tobacco (*N. tabacum* L.) cultivar K326, used as the WT, was provided by Yuxi Tobacco Seed Co., Ltd. Plants were grown under 14 h light /10 h dark at 28 °C/22°C, respectively.

### Cis-element analysis of *NtWRKY65* gene promoter

The promoter sequence (1.8 kbp upstream of the start codon) of the *NtWRKY65* gene (gene ID *Nitab4.5_0000437g0130.1* in the *N. tabacum* genomic annotation database [36]) was downloaded from the Sol Genomics Network (SGN) database (https://solgenomics.net/). Cis-elements in the promoter region were searched using PlantCARE web tools (http://bioinformatics.psb.ugent.be/webtools/plantcare/html/).

### Vector construction and transformation of *N. tabacum*

The *NtWRKY65* promoter sequence was amplified (forward primer: 5′-GAATTCGGTTAAACAAAGGGGTGAAA-3′, reverse primer: 5′-CATGCCATGGTGCAAGCAAATGTGAAACTAAT-3′) from the genomic DNA of K326 and inserted into pCAMBIA1381 vector to obtain a promoter-driven β-glucuronidase (GUS) construct.

The full-length coding sequence (CDS) of *NtWRKY65* was amplified (forward primer: 5′-ATGGAAGATTCAATAATATTT-3′, reverse primer: 5′-TCAACCCAAGGTCCCACAC-3′) from the cDNA of K326 leaves and inserted into pCAMBIA1305.1 vector to obtain the CaMV-35 S promoter driven overexpression construct.

The two constructs obtained as aforementioned were separately transformed into the *Agrobacterium tumefaciens* (strain GV3101) competent cells by using the freeze thaw method. The constructed vectors were transferred into K326 leaves by using the *Agrobacterium*-mediated method as previously described [59]. Briefly, 6-week-old K326 leaves were cut into small discs approximately 1 cm in diameter, and immersed into *Agrobacterium* solution (OD_600_ = 0.4–0.6), gently shaken for 8 min. After that, leaf discs were plated onto the regeneration medium (Murashige and Skoog [MS] medium contains 3% (w/v) sucrose, 1 mg/L 6-benzylaminopurine, 0.15 mg/L 1-naphthaleneacetic acid, 50 mg/L cefotaxime sodium, and 8 mg/L hygromycin, pH 5.6). The hygromycin resistant seedlings obtained were used for further analysis.

### GUS staining and GUS activity measurement

Tobacco seeds were sterilized in 70% ethanol, then grown in the soil until the three-leaf stage. Tobacco seedlings were treated with 5 mL of Hoagland’s solution containing 150 mM NaCl for 2 h, the normal Hoagland’s solution was set as the control. After that, the soil was carefully washed off the roots using distilled water.

For GUS staining, tobacco seedlings were incubated in GUS staining buffer (100 mM sodium phosphate, 10 mM EDTA, 1 mM 5-bromo-4-chloro-3-indolyl-β-D-glucuronic acid, and 0.5 mM potassium ferrocyanide) at 37 °C for 12 h, then destained in 70% ethanol for 12 h. Details on the methods are in the literature [60].

Quantitative determination of GUS activity was performed according to the previously described methods [61]. Briefly, leaves were homogenized by freezing with liquid nitrogen and ground using mortar and pestle, then the extraction solution (0.1% sodium lauryl sarcosine, 100 mM sodium phosphate (pH 7.0), 10 mM EDTA, 10 mM DTT, and 0.1% Triton X-100) was added. After centrifugation (12,000 g for 10 min at 4 °C), 100 µL supernatant was mixed with 400 µL reaction buffer containing 1 mM 4-methylumbelliferyl-β-glucuronide, and incubated at 37 °C for 30 min. The fluorescence was quantified using a fluorescence spectrophotometer (HITACHI F-4600, Tokyo, Japan) with excitation at 365 nm, emission at 455 nm. Protein concentration was determined using the Bradford method [62]. The GUS activity was normalized with 4-methylumbelliferone (MU) standards and expressed as nmol MU per minute per mg protein. Three seedlings were separately measured for each material.

### Subcellular localization assay

The CDS of *NtWRKY65* was inserted into pCAMBIA-2300-eGFP vector to generate CaMV-35 S::GFP-NtWRKY65 recombinant. The construct was transformed into *Agrobacterium tumefaciens* strain GV3101, then transiently transformed into the leaves of four-week-old tobacco plants (*Nicotiana benthamiana*) using previously described infiltration methods [63]. An empty vector was used as the control. The fluorescence signal of the fusion protein was observed using a laser confocal microscope (Leica SP8, Germany) 3 d after the injection.

### Transactivation assay

The CDS of the *NtWRKY65* gene was ligated into the pGBKT7 vector to fuse with the DNA-binding domain of *Saccharomyces cerevisiae* transcription activating protein GAL4 to obtain the pGBKT7-NtWRKY65 construct. pGBKT7-53 was used as the positive control, which encodes a fusion of murine p53 protein and the GAL4 DNA-binding domain, while pGBKT7 was used as the negative control. These plasmids were transformed into yeast strain AH109 (Clontech, USA), cultured on three types of medium (SD/-Trp, SD/-Trp with X-α-gal, and SD/-Trp/-His/−Ade with X-α-gal) at 30 °C for 2 d and transcriptional activation activity was detected based on the growth status and α-galactosidase activity.

### NaCl treatment and measurement of phenotype-related parameters

Tobacco seeds were sterilized and grown on MS medium for 2 weeks. The seedlings were transplanted into pots containing a mixture of vermiculite and nutrient soil (1:1). After seven weeks of growth, tobacco seedlings were watered with 20 mL of Hoagland’s solution containing 150 mM NaCl every 2 d, and finally for 14 d, the normal Hoagland’s solution was set as the control. Phenotypes were observed and photographed at the end of the treatment, and leaves were harvested and stored at -80 °C until use.

The maximum leaf area was measured using a grid paper of 1 × 1 mm. The maximum leaf area of three seedlings was measured separately for each material.

Tobacco leaf chlorophyll content was measured using a SPAD-502 chlorophyll meter model (KONICA MINOLTA, Japan) according to a previously described method [64], three seedlings were separately measured for each material.

Leaf electrolyte leakage was determined according to a previously described method [8]. Briefly, ten discs of 0.5 cm diameter were cut from the largest leaf of each seedling, washed three times with deionized water to remove electrolytes adhered on the surface. Then the discs were placed into 5 mL deionized water, incubated at 10 °C for 24 h. The electrical conductivity of the solution (EC1) was determined using a conductivity meter (DDS-307, Shanghai INESA Scientific Instrument, China). Then the samples were incubated at 95 °C for 20 min, cooled down to 25 °C and the final electrical conductivity (EC2) was measured. The electrolyte leakage (EL) was calculated as follows: EL = (EC1/EC2) × 100 (%). Three seedlings were separately measured for each material.

### Root morphology observation

Sterilized tobacco seeds were germinated and grown in vertical MS medium for 14 d, and then transferred to MS medium containing 0 and 150 mM NaCl, respectively, and root length was measured after 10 d. For each material, three seedlings were separately measured.

### Antioxidant activity

CAT, SOD, and POD activities and MDA content were measured using a CAT activity assay kit, SOD activity assay kit, POD activity assay kit, and MDA content assay kit (Solarbio, China), respectively, according to the manufacturer’s instructions. The O_2_^−^ and H_2_O_2_ contents in tobacco leaves were measured using the O_2_^−^ assay kit and H_2_O_2_ kit (Solarbio, China), respectively, according to the manufacturer’s instructions. For each assay, three seedlings were separately measured.

Histochemical staining of O_2_^−^ and H_2_O_2_ was performed using DAB and NBT, respectively, according to previously described procedures [65]. Briefly, discs of 1 cm diameter were cut from the largest leaf of each seedling, then stained with 50 mM sodium phosphate (pH 7.5) containing 0.2% NBT or 10 mM sodium phosphate (pH 6.5) containing 1 mg/mL DAB, then vacuumed for 10 min and incubated at 37 °C for 10 h under dark or light conditions, respectively. After that, the leaf discs were washed with ethanol, rinsed in boiling water for 10 min to remove chlorophyll, and then transferred into fresh ethanol. Finally, the stained leaves were photographed using a camera.

### Determination of Na^+^ and K^+^ content

Tobacco leaves and roots were separately sampled, roots were first rinsed five times using deionized water to remove the adhered ions. The sampled leaves and roots were then dried in an oven at 105 °C for 30 min, followed by overnight drying at 60 °C. After that, the samples are ground into powder and filtered through 60-mesh sieve. The Na^+^ and K^+^ contents were measured according to a previously described method [66]. Sample (0.4 g powder) was digested in an automatic digester (Auto Digiblock S60 Up, Lab Tech, USA), then dissolved into 50 mL ultrapure water. Finally, the Na^+^ and K^+^ contents were measured using an inductively coupled plasma optical emission spectrometer (Varian Medical Systems, USA). The samples from three seedlings were separately analyzed for the target ions.

### Quantitative real-time polymerase chain reaction (qRT-PCR)

Leaf RNA was extracted using the FastPure universal plant total RNA isolation kit (Vazyme, China), and the quality and quantity of total RNA was determined using a Nanodrop 2000 spectrophotometer (Thermo Fisher, USA) and agarose gel electrophoresis. First-strand cDNA was synthesized using the HiScript II 1st strand cDNA synthesis kit (Vazyme, China). The detailed PCR mixture components and reaction conditions were set according to a previous method [59].

### RNA-Seq analysis

The extracted leaf total RNA was sent to Biomarker Technologies Co., Ltd. (Beijing, China) for library construction, followed by sequencing using the Illumina NovaSeq6000 platform. The 150-bp paired-end raw data were first subjected to quality control. Clean reads obtained were mapped to the reference genome [36] using Hisat2 software [67], and identification and functional enrichment of differentially expressed genes (DEGs) was performed using BMKCloud (www.biocloud.net). For each material, three biological replicates were set, each replicate contained one seedling.

### Statistical analysis

The experiments were conducted using at least three biological replicates per sample. The data was analyzed by one-way analysis of variance (ANOVA) and Student’s *t*-test using IBM SPSS Statistics 19 software (IBM, New York, USA).

### Electronic supplementary material

Below is the link to the electronic supplementary material.

Supplementary Material 1

Supplementary Material 2

Supplementary Material 3

Supplementary Material 4

Supplementary Material 5

## Data Availability

All data generated or analyzed during this study are included in this published article (and its supplementary information files). The raw filtered RNA-seq data used in this article are available in the NCBI Sequence Read Archive (SRA) (https://www.ncbi.nlm.nih.gov/sra/) under BioProject accession: PRJNA622901.

## Abbreviations

CAT: Catalase
CDS: Coding sequence
DAB: Diaminobenzidine
DEGs: Differentially expressed genes
GO: Gene Ontology
GUS: β-glucuronidase
MDA: Malondialdehyde
NBT: Nitro blue tetrazolium
POD: Peroxidase
ROS: Reactive oxygen species
SOD: Superoxide dismutase
WT: Wild type
