# Supplementary material for: Overexpression of the WRKY transcription factor gene NtWRKY65 enhances salt tolerance in tobacco (Nicotiana tabacum)
Source: BMC Plant Biol. 2024 Apr 24;24:326. doi: 10.1186/s12870-024-04966-0 (PMC11040801; doi:10.1186/s12870-024-04966-0)
Supplement: Supplementary file 2 — Supplementary Material 2 [file 12870_2024_4966_MOESM2_ESM.pdf]

Overexpression of the WRKY transcription factor gene *NtWRKY65* enhances salt tolerance in tobacco (*Nicotiana tabacum*)

Xiaoquan Zhang[1], Yaxuan Zhang[1], Man Li[1], Hongfang Jia[1], Fengjie Wei[2], Zongliang Xia[1], Jianbo Chang[2*], Xuelin Zhang[3*], Zhaojun Wang[1*]

[1] College of tobacco science, Henan Agricultural University, Zhengzhou 450046, China

[2]Sanmenxia Branch of Henan Provincial Tobacco Corporation, Sanmenxia 472000, China

[3]College of Agronomy, Henan Agricultural University, State Key Laboratory of Wheat and Maize Crop Science, Zhengzhou 450046, China

[*] **Corresponding author:**

Jianbo Chang

E-mail: cjb2206@126.com;

Xuelin Zhang

Email: xuelinzhang1998@163.com;

Zhaojun Wang

E-mail: zjwang@henau.edu.cn;

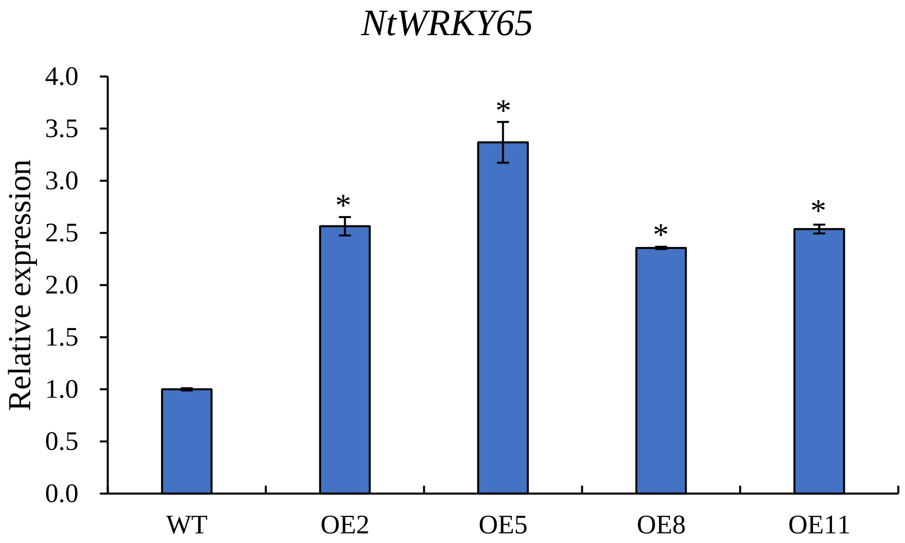

Fig. S1 Relative expression of *NtWRKY65* in overexpression lines and wild type plants. Gene expression level was measured by qRT-PCR, the columns and bars represent the means and standard errors (n = 3) respectively. The relative expression levels in different plants were calculated by setting the expression value in wild type plants as 1.

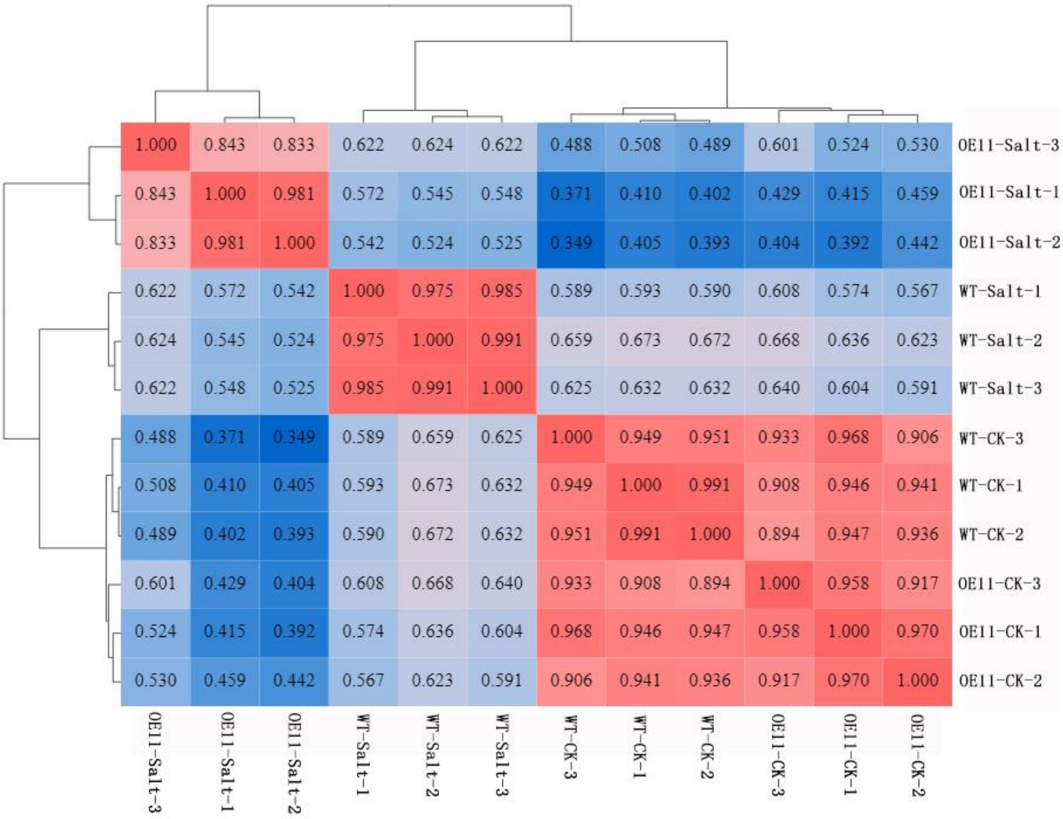

Fig. S2 Clustering of different RNA-Seq samples.
