# Supplementary material for: Overexpression of the WRKY transcription factor gene NtWRKY65 enhances salt tolerance in tobacco (Nicotiana tabacum)
Source: BMC Plant Biol. 2024 Apr 24;24:326. doi: 10.1186/s12870-024-04966-0 (PMC11040801; doi:10.1186/s12870-024-04966-0)
Supplement: Supplementary file 3 — Supplementary Material 3 [file 12870_2024_4966_MOESM3_ESM.docx]

Table S2 Summary of RNA-Seq reads for *NtWRKY65* overexpression line and wild type under 0 mM and 150 mM NaCl treatment

| Sample | Total Reads | Mapped Reads | Uniq Mapped Reads | Multiple Map Reads |
| --- | --- | --- | --- | --- |
| WT-CK-1 | 43,242,428 | 41,683,198 (96.39%) | 39,397,527 (91.11%) | 2,285,671 (5.29%) |
| WT-CK-2 | 43,760,368 | 42,194,091 (96.42%) | 39,930,013 (91.25%) | 2,264,078 (5.17%) |
| WT-CK-3 | 42,500,214 | 40,945,085 (96.34%) | 39,006,022 (91.78%) | 1,939,063 (4.56%) |
| OE11-CK-1 | 41,735,242 | 40,121,923 (96.13%) | 38,221,200 (91.58%) | 1,900,723 (4.55%) |
| OE11-CK-2 | 50,572,986 | 48,447,212 (95.80%) | 45,463,153 (89.90%) | 2,984,059 (5.90%) |
| OE11-CK-3 | 39,865,056 | 38,514,534 (96.61%) | 36,716,719 (92.10%) | 1,797,815 (4.51%) |
| WT-Salt-1 | 42,246,156 | 39,941,575 (94.54%) | 38,207,439 (90.44%) | 1,734,136 (4.10%) |
| WT-Salt-2 | 41,310,886 | 39,845,882 (96.45%) | 37,921,475 (91.80%) | 1,924,407 (4.66%) |
| WT-Salt-3 | 38,393,270 | 37,004,506 (96.38%) | 35,336,723 (92.04%) | 1,667,783 (4.34%) |
| OE11-Salt-1 | 42,034,432 | 39,406,728 (93.75%) | 37,728,014 (89.76%) | 1,678,714 (3.99%) |
| OE11-Salt-2 | 41,329,422 | 39,771,732 (96.23%) | 38,043,621 (92.05%) | 1,728,111 (4.18%) |
| OE11-Salt-3 | 41,843,616 | 39,998,516 (95.59%) | 38,175,125 (91.23%) | 1,823,391 (4.36%) |

Note: -CK and -salt in sample name mean 0 mM and 150 mM NaCl treatment, respectively. -1, -2 and -3 in sample name denote the biological replicates.
